# Nonspecific *Erysipelothrix rhusiopathiae* Bacteremia in a Patient with Subclinical Alcoholic Liver Disease

**DOI:** 10.1155/2013/474593

**Published:** 2013-05-30

**Authors:** Asim Ahmed Kichloo, Alexander Hallac, Ben Mousavi, Omkar Hirekhan

**Affiliations:** Department of Internal Medicine, Wyckoff Heights Medical Center, Brooklyn, NY 11237, USA

## Abstract

*Erysipelothrix rhusiopathiae*, a pleomorphic gram-positive bacillus, is found widely in nature or as a commensal pathogen. It infects domestic animals such as swine, which may be the major reservoir of the organism. *E. rhusiopathiae* is primarily an occupational illness; 89% of the cases are linked to high-risk epidemiological situations. Humans that are infected by this bacillus typically present with one or a combination of the following symptoms: localized skin lesion (erysipeloid), diffuse cutaneous eruptions with systemic symptoms, or bacteremia, which is often followed by endocarditis. We report a case of *E. rhusiopathiae* bacteremia that was present without severe clinical illness such as endocarditis, arthritis, or skin lesions. The patient was a 64-year-old male with a complicated past medical history including subclinical alcoholic liver disease. Penicillin-G therapy completely resolved the patients bacteremia. The case presented has exceptional clinical merit due to 2 key factors: the patient does not fit the occupational demographic typically affected by this bacterium, and the patient presented with subclinical septicemia, which has a high correlation with fatal endocarditis. This case brings a new prospective to *E. rhusiopathiae* bacteremia.

## 1. Introduction

*Erysipelothrix rhusiopathiae,* a pleomorphic gram-positive bacillus, is found widely in nature or as a commensal pathogen. It infects domestic animals such as swine, which may be the major reservoir of the organism. *Erysipelothrix rhusiopathiae* is also found in sheep, horse, cattle, chicken, crabs, dogs, and cats and is also encrusted in fish scales for long periods.

Infection in humans is mainly due to occupational exposure. Thus, abattoir workers, butchers, fishermen, farmers, and veterinarians are all at risk for infection.

## 2. Case

A 64-year-old male presented to the emergency department at Wyckoff Heights Medical Center. The patient had a past medical history of hypertension, diabetes mellitus type 2, bronchial asthma, depression, and chronic alcoholism. The patient's chronic alcoholism ended as per patient two weeks prior to admission. The patient denies current or previous drug abuse and tobacco abuse. Currently, the patient resides alone in a private residence where he cares for himself and has little social support. The patient previously worked at a children's toy manufacturer. There is no history of occupational exposure to live animals; however, the patient frequently purchases his poultry and fish from markets where the animals are kept alive. He presented to the ER with complaints of epigastric and left flank pain of 7 out of 10 severity that radiated to the back. The pain was reportedly accompanied by subjective fever for 5-6 days prior to hospitalization. Vital signs on presentations are as follows: temperature of 37.8°C, pulse 96 bpm, respiratory rate of 19 per minute, blood pressure of 143/74 mm of Hg, and pulse ox 96% on room air Leukocyte count on admission was 4.99 K/UL. The patient's epigastric and left flank pain was cramping in nature and not related to food intake. There were no aggravating or alleviating factors present. In addition to the pain, the patient was experiencing dysuria for 7 days but denied any change in urine color, burning micturition, hesitancy or urgency. He denied chills, rigors, night sweats, loss of appetite, chest pain, shortness of breath, or any additional constitutional symptoms. Physical exam findings showed hepatomegaly and no joint swelling or skin lesion. The cardiac examination included an EKG that showed an unremarkable sinus rhythm with premature ventricular complexes; cardiac auscultation revealed no murmurs.

The initial diagnostic interventions performed were X-ray of the lumbosacral spine (2-3 views) to rule out spinal pathology.

CT of the abdomen and pelvis was performed to rule out pancreatitis. The imaging studies revealed cirrhotic liver, splenomegaly with trace ascites, and varices. Finally, there was minimal stranding around the pancreatic tail that did not rule out acute pancreatitis.

Following up the unremarkable diagnostic images, an initial blood culture was collected on the first day of hospitalization and grew gram-positive rods. This initial culture was followed up with a more sensitive Kirby-Bauer diffusion disc method, which identified the presence of *Erysipelothrix rhusiopathiae*. Two repeat blood cultures preformed on the second day of hospitalization confirmed the bacteremia. On the fourth day of hospitalization, two cultures were obtained and grew *Erysipelothrix rhusiopathiae* clearly establishing the diagnosis.

The strong cardiac implications of this specific type of bacteremia required investigation by both an echocardiogram and a transesophageal echocardiogram (TEE). The echocardiogram was preformed first and revealed a normal left ventricular systolic function. Additionally, it also showed no mobile echodensities suggestive of infective endocarditis. Transesophageal echocardiogram revealed no evidence of mobile echodensities that are suggestive of infective endocarditis, in spite of the presence of trivial pericardial effusion along with trace tricuspid regurgitation, mild mitral regurgitation, and annular calcification. The findings of both diagnostic tests excluded the possibility of the *E. rhusiopathiae* involving the heart.

Laboratory results were as follows: total bilirubin 2.1, direct bilirubin 1.1, AST 164, ALT 40, albumin 4.0, total cholesterol 207, LDL cholesterol 148, HDL cholesterol 25, tumor marker AFP serial 11.5, C-reactive protein, cardio: 9.20 mg/L (0–10 mg/L), ESR: 90 mm/hr (0–14 mm/hr), and HIV test negative. In the labs above, the AST elevation ratio of >2 : 1 in relation to ALT shows positive proof of chronic alcoholism.

The plan of management of the patient's symptoms was to eliminate the bacteremia and exclude all possible disease sequelae of *E. rhusiopathiae*. After investigating all relevant laboratory and imaging studies, the definitive course of action was antibiotic therapy. *E. rhusiopathiae* was susceptible to Penicillin-G, Erythromycin, Ampicillin, and Gentamicin. Pharmacotherapy was initiated on day 3 of hospitalization and consisted of Penicillin-G 4 million units IVPB every 4 hours for a 4-week duration. This therapy resulted in complete resolution of the patient's *E. rhusiopathiae* bacteremia in 5-day time, which was supported by a negative blood culture on day 8 of hospitalization.

## 3. Discussion

*E. rhusiopathiae,* a gram-positive bacillus, was first isolated by Koch in 1878 and later identified as a pathogen by Rosenbach in 1909. *E. rhusiopathiae* is an occupational illness; 89% of the cases are linked to high-risk epidemiological situations [1]. Bacteremic infection, with or without endocarditis, is most commonly a primary infection rather than dissemination from localized cutaneous lesions. Approximately, one-third of patients with systemic infection do present with erysipeloid skin lesions suggestive of cutaneous contact [2–4]. Persistent bacteremia or endocarditis has been reported after ingestion or manipulation (occupational exposure) of contaminated seafood or undercooked pork [2, 3]. The clinical presentation of *E. rhusiopathiae* bacteremia may slightly resemble that of gram-negative sepsis. This bacteremia may be seen in patients with severe underlying liver disease. More than one-third of patients with systemic infection are alcohol dependent. Drug dependence, immunosuppression, and chronic liver disease are important predisposing factors. *E. rhusiopathiae* bacteremia is most commonly associated with severe clinical illness and is often complicated by endocarditis.

Here, we have a case where *E. rhusiopathiae* bacteremia was present without severe clinical illness such as endocarditis, arthritis, or skin lesions. The initial absence of typical clinical or echocardiograph features of endocarditis does not exclude the possibility in patients with positive blood cultures; therefore, a 2D echocardiogram was followed by a TEE, to rule out any vegetations in the heart.

Penicillin is the drug of choice for all forms of *E. rhusiopathiae* based on *in vitro* data. There are no clinical trials to date that confirm Penicillin's superiority in efficacy. *In vitro* Penicillin and Imipenem are the most active agents. Other active antibiotic agents include beta-lactam antibiotics, fluoroquinolones, clindamycin, daptomycin, and linezolid.

*E. rhusiopathiae* is typically resistant to sulfonamides, trimethoprim/sulfamethoxazole, vancomycin, and aminoglycosides.

In conclusion, *E. rhusiopathiae* is a rare organism that is causative of infective endocarditis. *E. rhusiopathiae* bacteremia/septicemia is coincidental with infective endocarditis in up to 90 percent of its patients, as per prior studies [4]. This case report draws attention to *E. rhusiopathiae* bacteremia in a patient with hepatic dysfunction due to alcoholic liver disease (cirrhosis). Chronic alcoholism is a known risk factor for *E. rhusiopathiae* infection [5]. There are very few studies and cases reported that present *E. rhusiopathiae* bacteremia without endocarditis in immunocompetent patients who have alcohol related hepatic damage.

An unclear history of the uncooked animal meat consumed by the patient and taking into account the fact that he is living alone are important diagnostic clues. As stated by the patient, he consumes a lot of fish (frozen) and pork not exactly knowing if it has been properly cooked or not. This points to the fact that patients who have cirrhosis can develop bacteremia with *E. rhusiopathiae* without any manifestations of endocarditis or other severe clinical manifestations.

Treatment for *E. rhusiopathiae* bacteremia is the same even if there is no cardiac involvement. The patient has to be treated for an adequate amount of time until the blood cultures are negative in order to prevent severe complications such as endocarditis. There have been no clinical studies to examine the long-term outcomes of patients with noncardiac *E. rhusiopathiae* bacteremia who go untreated.

There must be emphasis on the fact that this infection results primarily from occupational exposure or rarely from eating meat or fish that has been prepared incorrectly. Safe work practices and proper food preparation must be taught and emphasized to at risk individuals especially if they are immunocompromised or suffer from cirrhosis.
